# Income-related inequality and inequity in children’s health care: A longitudinal analysis using data from Brazil

**DOI:** 10.1016/j.socscimed.2019.01.040

**Published:** 2019-03

**Authors:** Anderson Moreira Aristides dos Santos, Julian Perelman, Paulo de Andrade Jacinto, Cesar Augusto Oviedo Tejada, Aluísio J.D. Barros, Andréa D. Bertoldi, Alicia Matijasevich, Iná S. Santos

**Affiliations:** a Postgraduate Program in Economics, Federal University of Alagoas, Brazil; b Centro de Investigação em Saúde Pública, Escola Nacional de Saúde Pública, Universidade Nova de Lisboa, Portugal; c Postgraduate Program in Economics, Federal University of Paraná, Brazil; d Postgraduate Program in Economics, Federal University of Pelotas, Brazil; e Postgraduate Program in Epidemiology, Federal University of Pelotas, Brazil; f Department of Preventive Medicine, Faculty of Medicine, FMUSP, University of São Paulo, Brazil

**Keywords:** Brazil, Children's health care, Inequality, Longitudinal data, Children's health

## Abstract

The Brazilian Unified Health System was created in the late 1980s to ensure free universal access to health care and was funded by taxes and social contributions. The persistent inequity in access to health services in favour of richer individuals in Brazil has been observed in the literature. However, to the best of our knowledge, no measurement of inequality in medicine use or private health insurance (PHI) among children has been performed with longitudinal data. This paper uses inequality indices and their decompositions to analyse the income-related inequalities/inequities in children's health care in the city of Pelotas, Brazil, using longitudinal data following children from 12 to 72 months of age. Our sample with data in all waves has between 1877 and 2638 children (varying according to outcome). We seek to answer three questions: i) How does the inequality/inequity in health care evolve as children grow up? ii) What are the main factors associated with inequality in children's health care? iii) How much of the change in inequality/inequity is explained by mobility in children's health care and income mobility? We found that inequities in health care have their beginnings in early childhood but that there was a reduction in inequity at 72 months of age. Ownership of children's PHI was associated with greater pro-rich inequity in health care. The reduction in inequality/inequity was linked to mobility in the sense that initially poorer children had greater gains in health care (a greater increase in PHI ownership and a lower reduction in medicine use). Despite this improvement among the poorest, apparently, the Brazilian public health service seems to fail to ensure equity in health care use among children, with possible long-term consequences on inequalities in health.

## Highlights

• The inequities in health care have their beginnings even in early childhood.
• Income and mother's education have a strong contribution in the inequalities.
• Private health insurance has strong contribution in inequalities of medicine use.
• There was reduction in inequity for children's health care in Pelotas/Brazil.
• This improvement for poorest children occurred when they reached 72 months.

## 1 Introduction

The Brazilian Unified Health System (SUS) was created in the late 1980s to ensure free universal access to health care and was funded by taxes and social contributions. The SUS included coverage for primary, secondary, and tertiary care; dental health services; prescription drugs; and diagnostic and therapeutic services in a decentralized model involving different spheres of federal, state, and municipal governments (Almeida et al., 2013).

Public spending on health as a proportion of Gross Domestic Product (GDP) increased sharply from 2.5% to 4% in the years since the creation of the SUS (1988), oscillating over time at this maximum level in the period between 1990 and 2010. However, this public spending is still much lower than that of OECD countries (Gragnolati et al., 2013). According to the WHO (2017), government health expenditures in Brazil as a proportion of GDP were 3.8% in 2014, and the percentage of public expenditures on total health expenditures was only 46%. Thus, although the SUS has allowed a significant increase in access, it ends up being insufficient, causing the private system to continue to develop and generating greater access to those who can obtain private health insurance or afford out-of-pocket expenses.

Private health insurance attracts a considerable share of the Brazilian population because it reduces waiting times and offers greater comfort (Santos, 2011; Santos et al., 2008). Private health expenditures have thus been growing in the last three decades (Almeida et al., 2013; Silveira et al., 2007), as a complement and duplication of the SUS (Santos et al., 2008). Since the private sector is accessible only to those who can afford private insurance (with or without co-payments) or can pay the full private price out-of-pocket, the existence of the private sector is potentially a major factor of inequity in the Brazilian health system, which contradicts the SUS equity principles. In other words, considering that the SUS is universal, individuals with access to health services through the private sector (PHI or out-of-pocket expenditures) have dual coverage. Andrade et al. (2018) argued that in 2013, 28% of the Brazilian population had PHI (data from the National Health Survey), and of the total number of people with this type of coverage, approximately 20% used services through the SUS, with emphasis on high complexity services, vaccinations, and emergencies. Furthermore, this study indicated low coverage for the medicines in the health services, whereby 64% of the individuals who consulted a doctor in the two weeks prior to the study acquired medications through out-of-pocket expenditures.

The persistent inequity in access to health services in favour of richer individuals in Brazil has been observed but is trending downward (Almeida et al., 2013; Andrade et al., 2013; Cambota and Rocha, 2015; Macinko and Lima-Costa, 2012). Macinko and Lima-Costa (2012) analysed health care utilization in Brazil from 1998 to 2008 and showed that doctor visits, visits to the dentist, and non-differentiated health services had a pro-rich concentration. Crucially, these authors showed that having private health insurance was an important factor associated with inequity. This result is no surprise in light of the widely demonstrated positive association between income and absolute health expenditures (Andrade et al., 2006; Garcia et al., 2013; Silveira et al., 2007, 2006).

Childhood is a very important stage of life, considering that poor health in childhood determines adult health and socioeconomic (SE) conditions (Case et al., 2002). If children from low SE backgrounds have less access to care, we can expect severe consequences for their present and future health. This is why there is a special interest in studying the inequity related to the use of private care in this specific category. The analysis of inequity in health care among children is a major step to understanding inequalities in health among adults. Furthermore, to the best of our knowledge, there is no measure or analysis of the index of inequality/inequity in health care (e.g., for medicine use) among children using longitudinal data, and the evidence in the international literature is scarce and based only on cross-section data (Layte and Nolan, 2015).

Indeed, although many researchers have used cross-sectional data for health care inequality analyses, few studies have relied on longitudinal data (Allin et al., 2011; Bago d’Uva et al., 2009). Cross-sectional data, even if collected over time, do not present all the advantages that longitudinal data can bring (Allanson et al., 2010). First, using data from children followed over time allows us to analyse the evolution of inequity as they grow older, and its determinants. Following cohorts over time helps to identify the ages at which children are more susceptible to inequity, which limits the risk of bias that would arise when comparing different ages and/or children through cross-sectional data. Second, as Jones and Nicolás (2004) and Allanson et al. (2010) explained well, cross-sectional analysis cannot capture some important features of a longitudinal analysis, namely, the mobility aspects. An extreme and clear example is when there is inequality in the initial period and in the next period, even though the children swap both health expenditures and income ranks. In this case, the inequity does not change despite the mobility. Cross-sectional analyses fail to account for such mobility (Chatterji et al., 2013).

In this paper, we analyse the income-related inequality and inequity in the health care of children and its trends as children grow older using longitudinal data including household health care costs, private health insurance, and medicine use. Based on a longitudinal sample from Pelotas, Brazil, this article seeks to answer three main questions: i) What is the magnitude of inequality/inequity in health care among children, and what is its evolution as they grow up? ii) What are the main factors associated with inequality in children's health care? iii) How much of the change in inequality/inequity is explained by mobility in children's health care and income mobility (ranking)? To the best of our knowledge, this is the first study to analyse income-related medicine use and insurance coverage for children using longitudinal data.

This paper is divided into four sections. Section 2 provides a brief summary of the database and methodology used in this paper. Section 3 shows the results. Section 4 concludes the paper.

## 2 Methods

Before explaining the methods in detail, it is worth summarizing the main steps of the analysis. We first analysed the evolution of inequality in health care variables using the concentration index (CI) and Erreygers’ index (EI) in four waves from 12 months to 72 months. We also examined the evolution in horizontal inequity (HI). Second, the CI (EI for binary variables) was decomposed into its determinants for each wave. Third, we decomposed the changes in the CI, EI, and HI into two mobility indices. These steps are detailed below.

### 2.1 Inequality and inequity measurements

First, we measured the income-related inequality in health care variables using the CI and EI. The CI is an indicator that is widely used in the literature (O’Donnell et al., 2008; Wagstaff et al., 1991), which takes on a positive (negative) value when there is pro-rich (pro-poor) inequality. We evaluated the evolution of the CI by measuring it in each wave. The CI is bounded between −1 and 1; however, when the variable of interest is binary, Wagstaff (2005) showed that these bounds are $\bar{h}$-1 and 1- $\bar{h}$ (where $\bar{h}$ is the mean of the outcome) and proposed a normalized CI, which is calculated by dividing the CI by (1-$\bar{h}$). Another approach is the normalization with a correction by Erreygers (2009), in which the CI is multiplied by 4 and $\bar{h}$. We applied this last normalization for our binary variables, which we represent by Erreygers' index (EI). We also applied the normalization provided by Wagstaff (2005), which gives similar results (these are available upon request). While the CI represents a relative index of inequality, EI is an absolute index of inequality. We will be using these two types of indices, the former for expenditure variables and the latter for binary variables (medicine use and PHI). It should be noted that the literature mentions that different indices are related to different vertical equity judgements (Allanson and Petrie, 2013; Kjellsson et al., 2015; Kjellsson and Gerdtham, 2013).

Then, we measured inequity in health care. That poorer children use less medicine and other health goods and services does not necessarily result in inequity (i.e., an unjust allocation), if their needs are also lower. The concept of inequity is related to a normative view of social justice, which was not present in the measurement of inequality. Most papers in health economics have adopted horizontal equity as the equity criterion, stating that people with equal health needs should be treated equally (Bago d’Uva et al., 2009; García-Gómez et al., 2015; Van Doorslaer et al., 2004; Wagstaff and Van Doorslaer, 2000).

We measured the inequity in health care using the horizontal inequity (HI), and it was also measured for each wave based on the concentration index of the needs-indirectly standardized outcome variable values given by the difference between the actual and need-predicted outcome plus the overall sample mean. When the HI is positive (negative), there is horizontal inequity in favour of the wealthier (poorer) child. These need-predicted outcomes were based on regression models. However, variables of health care expenditures are generally non-normally distributed and skewed, with a heavy right-hand tail, and in this study, these variables also have many zero values. Therefore, in order to consider a more adequate distribution, we ran regressions using generalized estimation equations (GEEs) with a log link, normal distribution family, and independent correlation for expenditure variables and random effects probit panel models for binary variables. This first model is equivalent to a GLM log-normal pooling. We first compared cross-sectional models through performance tests including the link test, Pearson correlation, Hosmer-Leme test, root mean squared error (RMSE) and mean absolute prediction error (MAPE). The GLM with a log normal distribution showed the best performance. Then, we ran GEE models with a log normal distribution, and our choice of correlation structure was based on the test proposed by Pan (2001) and its application in Stata software by Cui (2007).

The regression models included need and non-need variables (see Table 1), wave dummies (for controlling trends), and interactions between income and wave and between education and wave. These interactions seek to capture changes in the relationship between outcomes and explanatory variables.

**Table 1:** Description of variables.

Variable	Description
Outcomes	
Health Care	
Private Health Insurance	Dummy variable that equals one when the child has private health insurance
Medicine Use	Dummy variable that equals one when the child took medicines in the 2 weeks previous, including vitamin or medicine for fever.
Private Health Insurance Expenditures	Out-of-pocket expenditure (monthly premiums) on private health insurance (BRL).
Medicine Expenditures	Out-of-pocket expenditure for medicines on children in the 30 days previous to the interview (BRL).
Sum of Expenditures	Sum of out-of-pocket expenditure on medicines; private health insurance; Doctor's appointments; laboratory tests and r-rays; and others for children in the 30 days previous to the interview (BRL).
**Non-Need Variables**	
Income	Natural logarithm of income that is the sum of income (last month) of all household individuals divided by square root of household member number.
Asset Index	Construction of one index using principal components analysis (in quintiles at wave). Variables used (the child has in his household): vacuum cleaner; washing machine; DVD; refrigerator; microwave; computer; cell phone; TV; automobile; air conditioner.
Mother's Education	Mother's education in years.
Partner	Dummy variable that equals one when the mother lives with a partner
Mother's skin colour	Dummy variable, white = 1; brown, black, other = 0.
**Need Variables**	
**Children's Characteristics**	
Sex	Dummy variable, girl = 1; boy = 0.
Reported Health	Children's health reported by mother, categorical variable (excellent = 1, very good = 2, good = 3, regular and bad = 4).
Wheezing Chest	Child had wheezing in the Chest in the last 12 months: yes = 1, no = 0.
Chronic Disease	Dummy variable that equals one when the child has chronic diseases, physical problem or learning disability.
Low Birthweight	Dummy variable that equals zero when the child was born with a weight below 2500 g
Hospitalization	Dummy variable that equals one when the child was hospitalized during the first year of life.
Earache	Dummy variable that equals one when the child had an earache pain during the first year of life.
Pneumonia	Dummy variable that equals one when the child had pneumonia during the first year of life.
Urinary Infection	Dummy variable that equals one when the child had urinary infection during the first year of the life.
Breastfeeding Duration	Breastfeeding duration in months classified in following five categories: 0; 1 to 3; 4 to 5; 6 to 11; and 12 months or more.
**Mother's characteristics**	
Age	Mother's age in years.
Smoked during pregnancy	Dummy variable that equals zero when the child's mother smoked during the pregnancy.
Self-reported health	Mother's reported health, categorical variable (excellent = 1, very good = 2, good = 3, regular and bad = 4).

Notes: Mother's education was unavailable at wave 12 months. In this case, we used the values from the first wave (perinatal). Income and health expenditure variables were deflated for December 2011 (R$-BRL). Less than 1% of income values were zero, and they were transformed as 1 (R$) for the use of natural log. For mother's and child's reported health, the categories regular and bad were joined because the proportion of individuals responding bad is very small.

### 2.2 Decomposition of inequality

Second, the CI (EI for binary variables) was decomposed into its various components to determine the contribution of each health expenditure determinant to inequality, following the method proposed by Wagstaff et al. (2003). This decomposition allowed us to analyse how various factors contributed to a greater or lower inequality in health expenditures. Assuming a linear model, these authors observed that this index can be written as a weighted sum of CIs of explanatory variables. The contribution of each determinant is calculated by the product of the elasticity (impact on health expenditures of the determinant) and the income-related inequality of this determinant. Therefore, following the literature in the use of a linear approximation in decomposition of CI, we used the average marginal effect generated from non-linear models applying the same method used in the HI calculation. For this decomposition, when the binary variables were the outcomes, we used a similar approach, considering the concept and measurement of EI.

### 2.3 Decomposition of inequality and inequity changes into mobility indices

Third, for the longitudinal analysis of mobility, we used Allanson et al.'s (2010) approach, who proposed a decomposition of the change in the concentration index between two periods (initial s and f final) into two components: the income-related health mobility and health-related income mobility index. This change is represented by equation (1).

(1) $$\Delta CI=CI^{ff}-CI^{ss}=\left(CI^{ff}-CI^{fs}\right)-\left(CI^{ss}-CI^{fs}\right)=M^{R}-M^{H}$$

where *CI* is the concentration index, and its superscript term represents first the health care outcome variable and next the income ranking, both in f (final period, waves 24, 48, and 72 months) and s (initial period, wave 12 months) periods. In other words, *CI*^*fs*^ represents the concentration index when health care outcomes in the final period are ranked by income in the initial period.

*M*^*R*^ is the health-related income mobility, which measures the concentration index of final health care outcomes and changes in income rank. This mobility index can be positive or negative; it depends on whether the correlation between health care outcomes with the income rank is stronger in the initial period or in the final one. *M*^*H*^ is the income-related health care mobility. It is positive if changes in outcome variables are progressive. This is the case, for example, when the poorest children enjoy a larger share of total health expenditures in the final period compared to their initial share of health expenditures (or suffer lower losses). When *M*^*H*^ is negative, these changes in health expenditures are regressive. This index is equal to zero when there is no correlation between outcome and change income ranking or if the last term has no change.

This mobility index can be divided into two terms, where *p* represents the progressivity index (the CI of initial expenditures minus the CI of expenditure change both with rank income in initial period), and *q* is average expenditure change relative to an average expenditure of final period (equation (2)).

(2) $$M^{H}=\left(CI^{ss}-CI^{fs}\right)=\left(CI^{ss}-CI^{\Delta h,s}\right)\;\left(\frac{\bar{\Delta h}}{{\bar{h}}^{f}}\right)=p\;q$$

Furthermore, these two indices are also calculated in binary variables with Erreygers (2009) correction for the CI. Equations (1), (2)) can then be represented by equation (3) (Allanson and Petrie, 2013). The standard errors for longitudinal analyses indices are generated using bootstrapping.

$$\Delta EI=M_{EI}^{R}-M_{EI}^{H}=\left(EI^{ff}-\;EI^{fs}\right)-\left(EI^{ss}-\;EI^{fs}\right)=\left(EI^{ff}-\;EI^{fs}\right)-\left(\left(-CI^{\Delta h,s}\right)\;\left(4\bar{\Delta h}\right)\right),$$

then

(3) $$\Delta EI=M_{EI}^{R}-M_{EI}^{H}=\left(EI^{f,\;\Delta R}-p_{EI}q_{EI}\right)=EI^{f,\;\Delta R}+EI^{\Delta h,s}$$

where the indicators are as explained above, and the term "EI" indicates the application of correction for binary variables proposed by Erreygers (2009). The factor scale ($q_{EI}$) is calculated by multiplying the absolute changes mean ($\bar{\Delta h})$ by 4. The index progressivity ($p_{EI}$) is measured by negative term of the concentration index of health care changes ranked by initial income ($CI^{\Delta h,s})$. We can see that in this absolute index, for example, $M_{EI}^{H}$ depends on the relationship (covariance) between absolute changes in health care and the initial income ranking ($EI^{\Delta h,s})$.

We also measured M^R^ and M^H^ (of the CI and EI) for inequity analysis in similar ways but considering the concept and measurement of the HI, in which needs-standardized outcome replaces actual outcome. For all cases, we decompose (in mobility indices) the changes in the indices (the CI, EI, and HI) between two periods (waves), keeping 12 M as initial base wave.

### 2.4 Data

We used data from the 2004 Pelotas Birth Cohort conducted by the Center for Epidemiologic Research at the Federal University of Pelotas in southern Brazil. That cohort registered live births in the city of Pelotas and the neighbouring area of Jardim América district in the municipality of Capão do Leão in 2004. There were six follow-up visits at the following ages: perinatal, 3, 12, 24, 48 (4 years), and 72 months (6–7 years). In this last wave, children were between 6 and 7 years old; for simplicity, we say 72 months. All questionnaires related to all waves were approved by the Medical Research Ethics Committee of the Federal University of Pelotas affiliated with the Brazilian National Commission for Research Ethics (CONEP).

A total of 4231 children were born in the investigated area, with follow-up rates above 90% in the first five waves (Santos et al., 2011) and 88% at 72 months, so that 3799 children were assessed for a total of 4136 eligible (Barros et al., 2006; Santos et al., 2011).

### 2.5 Variables

Our analysis included five health care variables: 1) private health insurance (PHI); 2) medicine use; 3) out-of-pocket private health insurance expenditures (PHI expenditures); 4) out-of-pocket expenditures for medicine (medicine expenditures); and 5) total out-of-pocket health expenditures (total expenditures). The variables are available for four waves (12, 24, 48, and 72 M), except for medicine and total expenditures, for which data are not available for the 72 M wave.

The PHI variable indicates whether the child has private insurance or not, regardless of whether the family pays for it or whether it is offered by the employer of a family member. Furthermore, PHI expenditures measure the monthly health care payments paid directly by the family. Medicine use indicates if the child has taken medicine and/or vitamins in the last 15 days, regardless of whether they were obtained with payment or free of charge at public health units. Medicine expenditures measure the out-of-pocket payments for medicine in the last 30 days. Last, total expenditures are the total sum of out-of-pocket payments in the last 30 days (see Table 1). Furthermore, in the supplementary document, we also show measures for patient visits (wave 48 months) and the difficulty in obtaining a medical consultation (wave 12 months). Doctor visit variables were unavailable as longitudinal data.

Note that inequalities/inequities in private health expenditures and PHI do not necessarily indicate differences in use and access, since poorer children could be adequately accessing the public sector. However, the literature shows that PHI is strongly associated with inequality/inequity in the use of health goods and services (Santos, 2011; Santos et al., 2008). Furthermore, as a variable that is linked directly to health care use, we have medicine use for longitudinal data and consultations for cross-sectional data (for the last case, see the supplementary document).

Household income is the sum of the income of all household individuals divided by the square root of the household size, following the OECD equivalence scale (OECD, 2011). This variable and the health expenditure variables are deflated for December 2011 (R$-BRL). We used as a deflator the National Consumer Price Index (IPCA). This index covers families with monthly incomes ranging from 1 to 40 minimum wages of residents in urban areas. In addition, it is used as the official index of the inflation targeting regime. Many papers have used this index as a deflator (Garcia et al., 2013).

This paper follows the literature on the choice and classification of variables, in which non-need variables include income, race, and education, while sex, age, and health are classified as need variables (Van Doorslaer et al., 2004). All information was reported by the mother in most cases, except for birthweight and sex, which were obtained from birth records at the hospital.

We consider balanced panel samples for each expenditure variable analysis, given the availability of data. There are missing data in the variables, especially considering this balancing strategy, which were distributed as follows among the variables (accounting for the dropping out of children who did not participate in any wave): PHI (864), PHI expenditures (1827), medicine use (870), medicine expenditures (678), total expenditures (1427), income (1345) and all other variables (1237). In our final sample, the eligible percentages were as follows: 64% (PHI and medicine use), 76% (medicine expenditures), and 45% (PHI expenditures). Despite these significant losses, for an unbalanced panel and by dropping only the variables of interest, they were much smaller; for example, for PHI and medicine use, the final numbers were between 78% and 86% (varying according to wave). We also estimate an unbalanced panel in which the results are similar to those presented in this paper. The results are not shown but are available from the authors upon request. Another problem could be caused by selective mortality; however, only 2% of the children left the sample due to death.

All variables are detailed in Table 1, and a full description of the children's characteristics is available in Table A1.

## 3 Results

### 3.1 Evolution of inequality and inequity

**Fig. 1:**
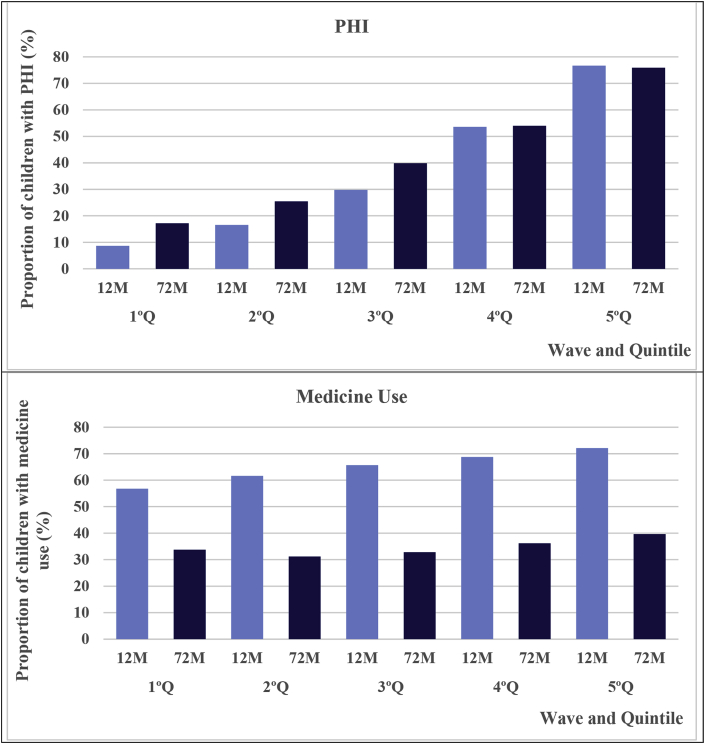
Proportion of children (%) with private health insurance (PHI) and medicine use by wave and quintile of initial income.

**Fig. 2:**
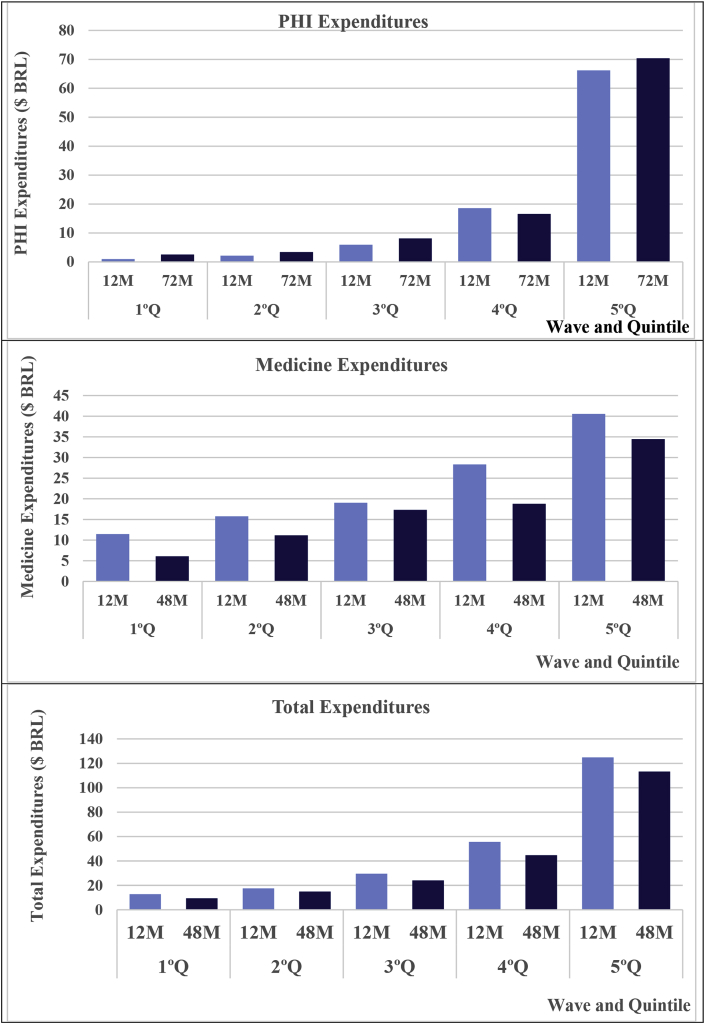
Mean of expenditures on children's health ($ BRL) by wave and quintile of initial income.

Fig. 1 shows the proportion of children with private health insurance (PHI) and those who used medicine in the last 15 days in the waves of 12 and 72 months by quintiles of initial income. The proportion of PHI strongly increased with income in both waves; however, the temporal growth of PHI was greater among the poorest children. The prevalence of medicine use was also related to income but to a much lower extent. This prevalence fell for all quintiles, but the decline was stronger among the richest. There was also a strong difference in expenditures on the health of children between the quintiles, which was favourable to the richest, in which we observed an increase in PHI expenditures for the poorest three quintiles and a reduction in medicine expenditures for all quintiles (Fig. 2).

The CI, EI, and HI, by wave, are shown in Table 2. In all cases, these indices are positive, indicating a pro-rich income-related inequality/inequity. There was an increase in the CI between 12 and 48 months for the total expenditures and medicine expenditures (note that for these indicators, no data were available for the wave 72 months). For PHI, PHI expenditures, and medicine use, EI and the CI were relatively stable in the first three waves but fell sharply in the 72-months wave. The analysis of HI indicated pro-rich inequities for all indicators. The evolution of this index was similar to the CI and EI, with strong decreases at 72 months for the indicators with data available at that date.

**Table 2:** Evolution of CI and HI, by outcome and wave.

	12 M	24 M	48 M	72 M
Concentration Index (CI)/Erreygers' Index (EI)				
Private Health Insurance (PHI)	0.572	0.589	0.554	0.484
N = 2638	(0.019)	(0.020)	(0.020)	(0.018)
Medicine Use	0.127	0.111	0.146	0.066
N = 2638	(0.021)	(0.023)	(0.021)	(0.021)
PHI Expenditures	0.681	0.674	0.662	0.572
N = 1877	(0.023)	(0.020)	(0.021)	(0.046)
Medicine Expenditures	0.255	0.224	0.326	–
N = 3145	(0.019)	(0.020)	(0.021)	–
Total Expenditures	0.475	0.464	0.517	–
N = 3145	(0.017)	(0.020)	(0.017)	–
**Horizontal Index (HI and HI**_**EI**_**)**				
Private Health Insurance (PHI)	0.528	0.535	0.501	0.435
N = 2638	(0.019)	(0.020)	(0.020)	(0.019)
Medicine Use	0.190	0.176	0.195	0.116
N = 2638	(0.020)	(0.021)	(0.023)	(0.020)
PHI Expenditures	0.662	0.655	0.643	0.549
N = 1877	(0.023)	(0.020)	(0.020)	(0.045)
Medicine Expenditures	0.324	0.294	0.385	–
N = 3145	(0.019)	(0.020)	(0.020)	–
Total Expenditures	0.486	0.480	0.524	–
N = 2509	(0.017)	(0.020)	(0.017)	–

Notes: EI and HI for binary variables were normalized by Erreygers (2009) approach. The standard errors of indices are in parentheses and were generated using bootstrapping with 300 replications. All values are statistically significant (p-values<0.01). Data are unavailable for medicine expenditures and total expenditures in the last wave (72 M).

Despite the similar conclusions for inequality and inequity trends, there was a wide difference in magnitude for medicine use. In this case, HI was higher than EI (both being positive), indicating that the poorest children generally take fewer medications/vitamins than wealthier children, although poor health is concentrated in these poorest children.

### 3.2 Decomposition of inequality

The percentage contributions of the groups of variables based on the first (12 M) and last waves of each outcome are presented in Table 3. Tables B1 to B3 contain more detailed results of the marginal effect and contribution of all variables. The income, asset index, and mother's education had the greatest pro-rich contribution to the CI and EI for wave 72 months. These three variables contributed to 92% of EI for PHI. Generally, need variables were not significant and had a small contribution to EI.

**Table 3:** Percentage Contribution (%) of variables to CI by outcome in the first and last wave.

	PHI	Medicine Use	PHI Expenditures	Medicine Expenditures	Total Expenditures
	12 M	12 M	12 M	12 M	12 M
Income/Asset Index	63.71	52.64	82.03	86.57	82.94
Health	−0.29	−45.42	−0.70	−32.66	−8.63
Mother's education	25.21	51.57	15.23	19.04	18.23
Need Others	5.65	−3.50	8.68	3.74	5.75
Non-Need Others	0.70	6.80	−4.09	6.07	−0.26
PHI	–	25.08	–	19.71	–
Residual	5.01	12.83	−1.16	−2.48	2.00
	**72 M**	**72 M**	**72 M**	**48 M**	**48 M**
Income/Asset Index	51.44	54.12	95.93	70.51	76.44
Health	−0.33	−66.26	−0.24	−20.61	−6.61
Mother's education	40.59	56.95	8.35	17.39	18.98
Need Others	7.33	−7.38	9.78	3.23	5.39
Non-Need Others	0.98	13.41	−5.20	4.84	−0.27
PHI	–	41.04	–	16.37	–
Residual	−0.01	8.12	−8.63	8.28	6.17

Notes: The groups correspond to the sum of percentage contributions of variables. **Income/Asset index** includes these two variables. **Health** includes reported health, wheezing chest, chronic disease, low birthweight, hospitalization, earache, pneumonia, and urinary infection. **Mother's Education** only includes mother's education. **Need Others** includes breastfeeding, mother's age, smoked during pregnancy, and sex. **Non-Need Others** includes mother's race and mother lives with a partner. **PHI** only includes private health insurance. The PHI variable was not included in the decomposition of total expenditures because this variable contains PHI expenditures in its composition.

Income/asset index and mother's education also had a strong contribution to EI of medicine use, 54% and 57%, respectively, for wave 72 months. Unlike the case of PHI, need variables had a strong contribution to EI in medicine use, in which children's health variables had a contribution of −66% for this index, i.e., poor people are sicker, and those with worse health in general are more likely take medicine. The private health insurance also contributed significantly (pro-rich) to inequality of medicine use (41%). We also analysed the CI for patient visits (wave 48 months) and the difficulty in obtaining a medical consultation (wave 12 months). There was inequality and inequity, and the PHI was a variable with a large contribution to inequality [Supplementary file C].

Income, asset index, and mother's education were also the variables that contributed the most to inequality in the CI of PHI expenditures. Expenditures for medicines presented results similar to medicine use, although with a lower contribution of the mother's education in detriment to a higher contribution of income/asset index.

Regarding changes in across waves, there was an increase in the relative contribution (%) the mother's education and a reduction in the relative contribution of income on EI of PHI. This situation was related to the higher and lower marginal effects of education and income, respectively. We also found a greater relative contribution of PHI and a more negative contribution of children's health on EI for medicine use. The major contribution of PHI to medicine use occurred along with an increase in the average of this variable; in other words, a greater proportion of children had a PHI, which occurred more commonly among the poorest.

### 3.3 Decomposition of inequality and inequity changes into mobility indices

Table D1 shows the decomposition of the changes between the waves 12 and 24 months in the CI, EI, and HI into the two mobility indices $M^{H}$ and $M^{R}$. ΔCI, ΔEI, and ΔHI are statistically non-significant [Supplementary file D]. Table 4 shows the decomposition of the changes between the waves 12 and 48 months in the CI, EI, and HI into the two mobility indices $M^{H}$ and $M^{R}$. In the case of medicine expenditures, there was an increase in inequality due to a disequalizing effect of M^H^. There was also an increase in inequality in medicine use, albeit small in magnitude and statistically non-significant; hence, this rise in inequality was possibly due more to differences in the amounts of out-of-pocket expenditures than to differences in the actual use of medicines. We observed reductions in PHI inequality and PHI expenditures, but these were statistically non-significant and small in magnitude.

**Table 4:** Decomposition of changes between 12 and 48 M waves in EI/CI and HI into mobility indices, by outcome.

	PHI	Medicine Use	PHI Expenditures	Medicine Expenditures	Total Exp.
ΔCI/ΔEI	−0.018	0.019	−0.019	0.071***	0.042**
	(0.022)	(0.031)	(0.022)	(0.026)	(0.018)
$M^{H}$	0.044**	−0.010	0.049***	−0.052**	−0.019
	(0.018)	(0.029)	(0.019)	(0.024)	(0.017)
p	0.201**	0.016	−6.768	0.168	0.117
	(0.099)	(0.047)	(102.487)	(0.104)	(0.134)
q	0.218***	−0.622***	−0.007	−0.311***	−0.166***
	(0.036)	(0.053)	(0.058)	(0.069)	(0.047)
$M^{R}$	0.026	0.009	0.030**	0.019	0.023*
	(0.016)	(0.018)	(0.015)	(0.015)	(0.013)
ΔHI	−0.026	0.005	−0.019	0.062**	0.038**
	(0.022)	(0.030)	(0.022)	(0.025)	(0.018)
$M_{HI}^{H}$	0.056***	0.002	0.050***	−0.039*	−0.014
	(0.018)	(0.029)	(0.019)	(0.023)	(0.017)
$M_{HI}^{R}$	0.029*	0.007	0.031**	0.022	0.024*
	(0.016)	(0.017)	(0.015)	(0.016)	(0.013)
N	2638	2638	1877	3145	2509

Notes: *, **, *** denotes p-values less than 10, 5, and 1%. ΔEI (for binary variables, PHI and Medicine Use), ΔCI, and ΔHI are respectively variations of EI, CI, and HI. M^H^ and M^R^ are income-related health care mobility and health-related income mobility for each of those cases. *p* represents the progressivity index and *q* is the factor scale. The standard errors of indices are in parentheses and were generated using bootstrapping with 300 replications. The subscript term HI represents the statistics for their respective cases.

Table 5 shows the decomposition of the changes between the waves 12 and 72 months in the CI, EI, and HI into the two mobility indices $M^{H}$ and $M^{R}$. First, all findings were similar to those obtained for inequality and inequity (EI and HI). For the cases of PHI and medicine use, the evidence was clearer for a significant decrease in EI (and HI). This change in both cases was formed almost entirely by an equalizing effect of income-related health care mobility ($M_{EI}^{H}$), in which there was an increase (absolute) in the proportion of children who had PHI ($q_{EI}$>0), and this increase was concentrated among the poorest ($p_{EI}$>0). Furthermore, the reduction in the prevalence of medicine use ($q_{EI}$<0) was concentrated among the richest children ($p_{EI}$<0). The findings for PHI expenditures were similar to PHI: there was an equalizing effect between the waves 12 and 72 months formed by a progressivity index (p) and relative changes (q) both being positive.

**Table 5:** Decomposition of changes between 12 and 72 M waves in EI/CI and HI into mobility indices by outcome.

	PHI	Medicine Use	PHI Expenditures
ΔCI/ΔEI	−0.089***	−0.062*	−0.109**
	(0.022)	(0.032)	(0.045)
$M^{H}$	0.084***	0.069**	0.050**
	(0.019)	(0.030)	(0.021)
p	0.388***	−0.057**	0.707
	(0.122)	(0.025)	(4.785)
q	0.217***	−1.210***	0.071
	(0.038)	(0.052)	(0.060)
$M^{R}$	−0.005	0.008	−0.059
	(0.018)	(0.019)	(0.047)
ΔHI	−0.092***	−0.074**	−0.114**
	(0.022)	(0.031)	(0.045)
$M_{HI}^{H}$	0.097***	0.080***	0.059***
	(0.020)	(0.029)	(0.022)
$M_{HI}^{R}$	0.005	0.006	−0.054
	(0.018)	(0.019)	(0.046)
N	2638	2638	1877

Notes: *, **, *** denotes p-values less than 10, 5, and 1%. ΔEI (for binary variables, PHI and Medicine Use), ΔCI, and ΔHI are respectively variations of EI, CI, and HI. M^H^ and M^R^ are income-related health care mobility and health-related income mobility for each of those cases. *p* represents the progressivity index and *q* is the factor scale. The standard errors of indices are in parentheses and were generated using bootstrapping with 300 replications. The subscript term HI represents the statistics for their respective cases.

## 4 Discussion

### 4.1 Key findings

This study analysed inequalities and inequities in children's health care using longitudinal data from the city of Pelotas in Brazil. To the best of our knowledge, no measurement of inequality in medicine use or PHI among children has been performed with longitudinal data. First, we observed pro-rich inequality and inequity in PHI, PHI expenditures, medicine use, medicine expenditures, and total health expenditures. However, there were reductions in inequality and inequity when children reached 72 months of age. Second, the variables that most contributed to pro-rich inequality of PHI and PHI expenditures were income, asset-index, and mother's education; for medicine use and expenditures, the variables that contributed the most to inequality were income, asset index, mother's education, PHI (pro-rich), and children's health (pro-poor). Third, the reduction in inequity in the last wave was largely explained by the greater increases in health care use (higher increase in PHI and a lower reduction in medicine use) among the poorest, in comparison with the first wave.

### 4.2 Interpretation

The SUS aimed at ensuring an integral, universal, and free public health system in Brazil, calling for equity in health care. However, earlier studies showed inequities largely associated with the existence of private health insurance (Almeida et al., 2013; Macinko and Lima-Costa, 2012; Mullachery et al., 2016; Santos et al., 2008). The case of inequity among children, which we analysed in this study, is of special relevance because a child's health is related to socioeconomic status and health in adulthood (Case et al., 2002).

First, despite the existence of a universal tax-financed health care system, our findings show that there is inequity in children's health care. Other studies in the literature report the existence of inequities in health care use for adults in Brazil (Almeida et al., 2013; Macinko and Lima-Costa, 2012; Mullachery et al., 2016). Garcia et al. (2013) show that there are inequalities in medicine expenditures in favour of higher income families in Brazil. In regard to inequity among children, the literature is scarce and hardly comparable. Let us mention the little or no inequity in GP visits observed in Scottish children (Layte and Nolan, 2015). This may be due to a lower weight of the private sector in the health sector in this country than in Brazil.

Second, consistent with our results, the literature shows the importance of income and mother's education to PHI (Cameron et al., 1988; Doiron et al., 2008; Johar et al., 2011; Silveira et al., 2007). The contribution of income is derived naturally from the fact that private insurance is a normal good (Doiron et al., 2008). The role of mothers' education can possibly be explained by greater health literacy and awareness, in addition to better socioeconomic conditions that allow access to insurance. In addition, the absence of an association between PHI and needs can be explained by the fact that people purchase private insurance preventively and not due to their current clinical status (Cameron et al., 1988). We showed that in addition to income, asset-index, and mother's education, PHI also has a strong pro-rich contribution to EI (CI) in medicine use (in addition to its effect on medical consultations and difficulty obtaining the consultations, see supplementary document).

Note that inequity in PHI could only indicate that richer people access the private system more frequently, while poorer individuals are covered by public sector services. In other terms, these results would not be a major concern if poor people are adequately covered by the public service. A study performed in Spain, where the system is also characterized by a duplicate private sector, showed pro-poor inequity in health care use within the public system for GP visits and hospitalizations (Regidor et al., 2008). The authors argued that the pro-rich inequity in the private sector was compensated for by the reverse phenomenon in the public one so that inequity should be examined simultaneously in both sectors: the richer use private services, while the poorer use public services. This result may also hold for Brazil; indeed, the literature shows that the SUS provides significant coverage and highlights some initiatives, such as the increasing participation of the Family Health Strategy (a programme based mainly on primary care and prevention in a decentralized way with health centred directly on communities). Macinko and Lima-Costa (2012) and Mullachery et al. (2016) indicated that the Family Health Strategy had a pro-poor relationship with inequalities in doctor visits and dental visits. Between 2002 and 2011 (the period covered by the analysis of this article), this programme increased coverage from approximately 40%–60% (Pinto et al., 2018). The 2000s were also marked by the creation of the National Emergency Care Policy, which had the main objective of guaranteeing universality, equity, and integrality in attending clinical, surgical, gynaecological-obstetrical, psychiatric, and paediatric emergencies and those related to causes. In institutional terms, during the same decade, an attempt was made to encourage the decentralization of the public health system; however, it is still a challenge to consolidate an efficient regionalized structure (Andrade et al., 2018).

From an ethical viewpoint, however, this question is controversial. Libertarians postulate that the ethical goal is to achieve minimum standards of care for everyone (the “decent basic minimum”, see (Pereira, 1993)), on the basis of rich people's altruism, i.e., the well-being of the poor enters the rich's utility function. Conversely, egalitarians are concerned that “the delivery of health care is organized in such a way that everyone enjoys the same access to care and that the care is allocated on the basis of need with a view to promoting equality of health” (Wagstaff and van Doorslaer, 2000b). There are several arguments to support this last statement, such as the fact that discrimination or exclusion of specific groups in health care is not acceptable. The other well-known argument, by the Nobel prize Amartya Sen, is that people should be offered the same “capabilities” to achieve good health and that health services are such a basic commodity to achieve good health and therefore good functioning (Pereira, 1993). Thus, the equity principle is that there should be “equality of basic capabilities”, i.e., the notion that also poor people should be entitled to access private insurance if they wish.

Furthermore, the “minimum standard of care” would hold if the Brazilian system was not a two-tier one. The public health service (SUS) only accounts for 41% of total health spending (much lower than in Western Europe countries, the USA or Canada), and the coverage of the Family Health Programme (primary care) is not complete, such as drug coverage. Paim et al. (2011) mentioned that “provision of secondary care by the SUS is problematic, because service supply is restricted and often given preferentially to individuals with private health plans” (p. 13). This would be an explanation for why there is a large inequity in health care use as a whole, without distinguishing the public and private sectors. Thus, there is a concern that the quality of care experienced by poorer people who do not have access to private insurance is lower than that offered to richer people in the private sector hence reinforcing the argument that access to PHI is an equity issue.

Finally, need variables have an important association with medicine use, as expected from the literature (Carrasco-Garrido et al., 2009; Oliveira et al., 2012; Santos et al., 2009); furthermore, they make a strong contribution to the CI. This result is as expected, given that children with poorer health should use more medicine, and poor health is concentrated in the poorest children.

Third, we sought to verify how changes in inequalities and inequities were related to two types of mobility: i) one would be mobility in health care, in which initially poorer children could have greater gains or lower losses in health care; ii) there is also a possibility of income mobility, in which there may be a relationship between changes in individuals positions in the income distribution and health care in the final period. Our results showed that the reduction in inequality/inequity was linked to health care mobility in the sense that initially poorer children had a greater increase in PHI ownership and a lower reduction in medicine use.

The main possible explanation for this health care mobility for PHI is that the living conditions of the poorest improved during this specific period. Indeed, we noted that the growth of incomes of the poor was much greater than that of the richest. Furthermore, studies have shown that in the 2000s, there were important income gains for the poorest in Brazil, leading to reductions in poverty and income inequality (Souza, 2012). The increase in the income and employment of the poorest may have enabled greater access to PHI. These results are also consistent with additional findings that showed a positive relationship between variations in income ranking and PHI ownership and a negative correlation between initial income and variations in income ranking.

In the case of medicine use, improvements in socioeconomic conditions may have benefited the poorest, in addition to leading to a higher proportion of children having health insurance. In addition, public policies may have benefited the poorest children; unfortunately, our data do not allow us to analyse the source of acquisition of these medicines. Finally, we also found a decline in the use of medicine as children grew up. The literature shows that younger children use more medicine, the prevalence decreases until adolescence and grows into adulthood, with high prevalence among the elderly (Arrais et al., 2005; Oliveira et al., 2012). This decrease was weaker for the initially poorest individuals, verifying a kind of convergence, which implied a reduction in inequalities and inequities. It should be noted that some episodes of poor health, such as wheezing in the chest, had a lower prevalence as children grew up.

### 4.3 Limitations

This study has some limitations. First, self-reported information may contain measurement errors, especially regarding expenditure variables. The survey questions relate to the period of the last 30 days, possibly introducing a recall bias problem. However, there is no evidence of a socioeconomic gradient in this recall bias (Ritter et al., 2001; Wolinsky et al., 2007), especially for this short period. In the case of medicine use in the last 15 days, interviewers asked the mothers to bring the medicine packages and prescriptions. Therefore, we expect that this limitation did not affect our results.

Second, the sample was composed of live births in the city of Pelotas in Brazil in 2004. This sample is, therefore, not nationally representative. Pelotas has a human development index greater than that of the country. However, our database contains relevant information with longitudinal data, and this advantage, compared to the use of a representative cross-section sample, off-sets the lack of representativeness.

Third, problems of endogeneity may arise due to reverse causality and/or omitted variables. We hope that the case of reverse causality between health care and income will not be strong for children, as this relationship is expected to be important in adulthood. Omitted variables may exist, but our database is relatively rich in socioeconomic information, maternal health, child health among other variables, which is a fact that minimizes this problem for the present article. However, we must indicate that due to possible problems of endogeneity, the effects found were not necessarily causal effects.

Fourth, there are some variables that were unavailable for the total period. For example, we observed an increase in the inequality of medicine expenditures between the 12 M and 48 M waves; however, the unavailability of data precluded us from concluding that, similar to medicine use, there was a reduction in the inequalities of these expenditures. Furthermore, doctor visit variables that were unavailable as longitudinal data could provide important information for our conclusions. However, we analysed medical consultations with cross-sectional data, and our results pointed to the same conclusions. Finally, the analysis was limited to children aged up to 6 and 7 years old (wave 72 months).

## 5 Conclusion

We found that despite the existence of a public health service, the inequalities and inequities in health care have their beginnings even in early childhood in Brazil. As children from low SE backgrounds have less access to health care, we can expect severe consequences on their present and future health. Even though the poorest children have worse health statuses, they have less health care than the richest children. Ownership of children's private health insurance is associated with greater pro-rich inequity in health care (use of medication and medical appointments). Therefore, richer children can have greater access, which can be obtained mainly through PHI and/or out-of-pocket, generating inequities. However, there was a reduction in the inequality/inequity with mobility in the sense that initially poorer children had greater gains in health care (a higher increase in PHI ownership and a lower reduction in medicine use). There was evidence that the poorest individuals had strong improvements in their socioeconomic conditions, such as income growth. These improvements in socioeconomic conditions may have implied greater access to health care.

Therefore, the SUS public policies that primarily benefit the poorest individual, such as the Health Family Strategy, are of fundamental importance. Public policies primarily focused on increasing children's access to health care can have a positive impact on children's health and therefore can bring benefits to these children in their adult lives. In this case, the public system should still seek equity that could be achieved with a stronger, more efficient and effective SUS.

In this case, inequalities/iniquities, for example, in the case of medicine use, have shown to be stronger before children turn 6 years old. Public policies that take this information into account may have better efficiency. Regarding the relevant socioeconomic factors that explain these inequities, in addition to income, the variable of the mother's education was shown to be of fundamental importance; this implies that reducing the gap between the mother's education among richer and poorer children can be an important measure in reducing these inequalities.

Given the limitations of this paper, future work may explore new data when children are older to yield results that can bring new information. Furthermore, important future research may seek to understand the factors associated with mobility by measuring the decomposition of income-related health care mobility into socioeconomic variables.

## Appendix A Supplementary data

A A

B B

C C

D D
