# Supplementary material for: Income-related inequality and inequity in children’s health care: A longitudinal analysis using data from Brazil
Source: Soc Sci Med. 2019 Mar;224:127–37. doi: 10.1016/j.socscimed.2019.01.040 (PMC6411923; doi:10.1016/j.socscimed.2019.01.040)
Supplement: A [file mmc1.docx]

**Supplementary document**

In this supplementary document, we seek to show some more detailed results. Table A1 shows the average of the explanatory variables for waves 12M and 72M per quintile of initial income (wave 12M).

**Table A1**. Mean of variables, by wave and quintile of initial income

|  | | **1º Quintile** | | **3º Quintile** | | **5º Quintile** | |
| --- | --- | --- | --- | --- | --- | --- | --- |
|  | **12M** | **72M** | **12M** | **72M** | **12M** | **72M** |  |
| **Outcome** | |  |  |  |  |  |  |
| PHI | | 0.087 | 0.172 | 0.298 | 0.398 | 0.767 | 0.759 |
| Medicine Use | | 0.568 | 0.338 | 0.656 | 0.328 | 0.721 | 0.400 |
| PHI Expenditures | | 0.977 | 2.549 | 5.934 | 8.124 | 66.209 | 70.391 |
| Medicine Expenditures | | 11.460 | 6.097 | 19.041 | 17.316 | 40.546 | 34.449 |
| Total Expenditures | | 12.831 | 9.361 | 29,505 | 24.013 | 124.835 | 113.214 |
| **Non-Need variables** | |  |  |  |  |  |  |
| Income | | 155.875 | 432.967 | 433.926 | 703.217 | 1730.921 | 2067.769 |
| Asset Index(1Q) - base | | 0.609 | 0.560 | 0.222 | 0.201 | 0.013 | 0.025 |
| Asset Index(2Q) | | 0.198 | 0.219 | 0.207 | 0.218 | 0.032 | 0.032 |
| Asset Index(3Q) | | 0.134 | 0.136 | 0.231 | 0.262 | 0.097 | 0.101 |
| Asset Index(4Q) | | 0.047 | 0.074 | 0.247 | 0.243 | 0.211 | 0.309 |
| Asset Index(5Q) | | 0.011 | 0.011 | 0.093 | 0.076 | 0.647 | 0.533 |
| Mother education (years) | | 5.925 | 6.158 | 7.932 | 8.397 | 11.436 | 12.133 |
| Mother live with Partner | | 0.777 | 0.779 | 0.822 | 0.806 | 0.917 | 0.861 |
| Mother skin color(white) | | 0.606 | 0.606 | 0.700 | 0.700 | 0.877 | 0.877 |
| **Need: Children's variables** | |  |  |  |  |  |  |
| Sex (female) | | 0.472 | 0.472 | 0.505 | 0.505 | 0.465 | 0.465 |
| Excellent Child's Health – base | | 0.281 | 0.270 | 0.427 | 0.359 | 0.539 | 0.427 |
| Very Good Child's Health | | 0.198 | 0.155 | 0.188 | 0.239 | 0.264 | 0.323 |
| Good Child's Health | | 0.421 | 0.472 | 0.307 | 0.355 | 0.173 | 0.230 |
| Regular or Bad Child's Health | | 0.100 | 0.104 | 0.078 | 0.047 | 0.025 | 0.021 |
| Wheezing Chest | | 0.713 | 0.260 | 0.647 | 0.241 | 0.507 | 0.182 |
| Chronic Disease | | 0.060 | 0.128 | 0.080 | 0.146 | 0.061 | 0.131 |
| Low Birthweight | | 0.091 | 0.091 | 0.093 | 0.093 | 0.066 | 0.066 |
| Hospitalization | | 0.260 | 0.260 | 0.167 | 0.167 | 0.121 | 0.121 |
| Earache | | 0.492 | 0.492 | 0.495 | 0.495 | 0.362 | 0.362 |
| Pneumonia | | 0.121 | 0.121 | 0.101 | 0.101 | 0.061 | 0.061 |
| Urinary Infection | | 0.049 | 0.049 | 0.046 | 0.046 | 0.061 | 0.061 |
| Breastfeeding (0) – base | | 0.092 | 0.092 | 0.110 | 0.110 | 0.101 | 0.101 |
| Breastfeeding (1-3 m) | | 0.153 | 0.153 | 0.125 | 0.125 | 0.082 | 0.082 |
| Breastfeeding (4-5 m) | | 0.170 | 0.170 | 0.171 | 0.171 | 0.175 | 0.175 |
| Breastfeeding (6-11m) | | 0.130 | 0.130 | 0.180 | 0.180 | 0.294 | 0.294 |
| Breastfeeding (>12) | | 0.455 | 0.455 | 0.414 | 0.414 | 0.349 | 0.349 |
| **Need: Mother's variables** | |  |  |  |  |  |  |
| Mother Age (years) | | 27.008 | 32.680 | 26.777 | 32.472 | 29.981 | 35.711 |
| Smoked in pregnancy | | 0.402 | 0.402 | 0.271 | 0.271 | 0.110 | 0.110 |
| Excellent Mother's Health – base | | 0.136 | 0.113 | 0.195 | 0.118 | 0.338 | 0.220 |
| Very Good Mother's Health | | 0.098 | 0.094 | 0.169 | 0.175 | 0.254 | 0.268 |
| Good Mother's Health | | 0.532 | 0.489 | 0.491 | 0.482 | 0.336 | 0.404 |
| Regular or Bad Mother's Health | | 0.234 | 0.304 | 0.144 | 0.226 | 0.072 | 0.108 |
| **N- observations** | | 530 | 530 | 527 | 527 | 527 | 527 |

Notes: Statistics based on the sample of outcomes of PHI and medicine use (except for health expenditures variables). For more details about the measure of variables, see table I.
