# Supplementary material for: Income-related inequality and inequity in children’s health care: A longitudinal analysis using data from Brazil
Source: Soc Sci Med. 2019 Mar;224:127–37. doi: 10.1016/j.socscimed.2019.01.040 (PMC6411923; doi:10.1016/j.socscimed.2019.01.040)
Supplement: B [file mmc2.docx]

**Supplementary document**

In this supplementary document, we seek to show some more detailed results. The Tables B1, B2, and B3 in the appendix detail the marginal effect of each explanatory variable and its contribution to the Concentration Index/ Erreygers’ Index (CI/EI). The percentage of contribution of each explanatory variable to inequality is presented in Figure B1.

**Table B1.** Marginal effects (M. E.) and contributions of need and non-need variables to EI of outcomes PHI and medicine use

|  | **M. E.- PHI** | | **Contribution-PHI** | | **M. E.- Medicine Use** | | **Contribution-Medicine Use** | |
| --- | --- | --- | --- | --- | --- | --- | --- | --- |
|  | **12M** | **72M** | **12M** | **72M** | **12M** | **72M** | **12M** | **72M** |
| **Non-Need Variables** |  |  |  |  |  |  |  |  |
| Income | 0.125*** | 0.061*** | 0.250 | 0.121 | 0.023*** | 0.009 | 0.046 | 0.017 |
| Asset Index(2Q) | 0.026* | 0.031* | -0.004 | -0.005 | 0.033** | 0.033** | -0.005 | -0.005 |
| Asset Index(3Q) | 0.094*** | 0.112*** | -0.003 | -0.003 | 0.035** | 0.035** | -0.001 | -0.001 |
| Asset Index(4Q) | 0.138*** | 0.165*** | 0.025 | 0.040 | 0.020 | 0.020 | 0.004 | 0.005 |
| Asset Index(5Q) | 0.194*** | 0.232*** | 0.096 | 0.095 | 0.047** | 0.047** | 0.023 | 0.019 |
| Mother’s education (years) | 0.033*** | 0.042*** | 0.144 | 0.196 | 0.015*** | 0.008** | 0.066 | 0.037 |
| Mother live with Partner | 0.000 | 0.000 | 0.000 | 0.000 | -0.010 | -0.010 | -0.001 | -0.001 |
| Mother’s skin color (white) | 0.018 | 0.022 | 0.004 | 0.005 | 0.043*** | 0.043*** | 0.010 | 0.010 |
| Private Health Insurance |  |  |  |  | 0.056*** | 0.056*** | 0.032 | 0.027 |
| **Need: Children's variables** |  |  |  |  |  |  |  |  |
| Sex (female) | -0.028** | -0.034** | 0.000 | 0.001 | -0.016 | -0.016 | 0.000 | 0.001 |
| Very Good Health | -0.009 | -0.011 | -0.001 | -0.001 | 0.071*** | 0.071*** | 0.004 | 0.009 |
| Good Child's Health | 0.001 | 0.001 | 0.000 | 0.000 | 0.136*** | 0.136*** | -0.028 | -0.025 |
| Regular or Bad Child's Health | -0.024 | -0.029 | 0.002 | 0.002 | 0.285*** | 0.284*** | -0.019 | -0.019 |
| Wheezing Chest | 0.014 | 0.017 | -0.002 | -0.001 | 0.067*** | 0.067*** | -0.011 | -0.004 |
| Chronic Disease | -0.005 | -0.005 | 0.000 | 0.000 | 0.112*** | 0.111*** | -0.001 | -0.001 |
| Low Birthweight | 0.012 | 0.014 | 0.000 | 0.000 | 0.010 | 0.010 | 0.000 | 0.000 |
| Hospitalization | -0.011 | -0.013 | 0.001 | 0.001 | 0.007 | 0.007 | -0.001 | 0.000 |
| Earache | 0.007 | 0.008 | -0.001 | -0.001 | 0.027** | 0.027** | -0.003 | -0.003 |
| Pneumonia | 0.018 | 0.021 | 0.001 | 0.001 | -0.004 | -0.004 | 0.000 | 0.000 |
| Urinary Infection | 0.077** | 0.092** | -0.001 | -0.001 | 0.048** | 0.048** | 0.000 | 0.000 |
| Breastfeeding (1-3 m) | -0.037 | -0.044 | 0.002 | 0.002 | -0.021 | -0.021 | 0.001 | 0.001 |
| Breastfeeding (4-5 m) | 0.011 | 0.014 | 0.000 | 0.000 | -0.030 | -0.030 | 0.000 | 0.000 |
| Breastfeeding (6-11m) | 0.026 | 0.031 | 0.003 | 0.004 | -0.025 | -0.025 | -0.003 | -0.003 |
| Breastfeeding (>12) | -0.028 | -0.034 | 0.002 | 0.002 | -0.028 | -0.028 | 0.002 | 0.002 |
| **Need: Mother's variables** |  |  |  |  |  |  |  |  |
| Mother’s Age (years) | 0.004*** | 0.005*** | 0.01 | 0.008 | -0.001 | -0.001 | -0.002 | -0.001 |
| Smoked in pregnancy | -0.062*** | -0.075*** | 0.014 | 0.015 | 0.016 | 0.016 | -0.004 | -0.003 |
| Very Good Mother's Health | 0.035** | 0.041** | 0.004 | 0.006 | 0.024 | 0.024 | 0.003 | 0.004 |
| Good Mother's Health | 0.014 | 0.017 | -0.002 | -0.001 | -0.008 | -0.008 | 0.001 | 0.000 |
| Regular or Bad Mother's Health | 0.010 | 0.012 | -0.001 | -0.002 | 0.025 | 0.025 | -0.003 | -0.005 |
| **residual** |  |  | 0.029 | 0.000 |  |  | 0.016 | 0.005 |
| **N- observations** | 2,638 | | | | 2,638 | | | |

Notes: *, **, *** denotes p-values less than 10, 5, and 1%. The base groups for asset index, child’s health, breastfeeding, mother’s health are: 1º quintile (1Q), excellent, zero months, and excellent. Estimations were realized with random effect probit model and include the interaction between wave 72 months and income, and wave 72 months and mother’s education. Contributions to CI were normalized by Erreygers’ (2009) approach (EI).

**Table B2.** Marginal effects (M. E.) and contributions of need and non-need variables to CI of outcomes PHI expenditures (PHI Exp.) and medicine expenditures (medicine exp.)

|  | **M. E.-PHI Exp.** | | **Contribution-PHI Exp.** | | **Marg. Effect-Medicine Exp.** | | **Contribution- Medicine Exp.** | |
| --- | --- | --- | --- | --- | --- | --- | --- | --- |
|  | **12M** | **72M** | **12M** | **72M** | **12M** | **48M** | **12M** | **48M** |
| **Non-Need Variables** |  |  |  |  |  |  |  |  |
| Income | 10.164*** | 11.409*** | 0.274 | 0.285 | 4.783*** | 3.965*** | 0.107 | 0.107 |
| Asset Index(2Q) | 9.494 | 10.657 | -0.009 | -0.026 | 5.987*** | 4.963*** | -0.009 | -0.011 |
| Asset Index(3Q) | 15.476** | 17.371** | -0.014 | -0.012 | 10.449*** | 8.663*** | -0.003 | -0.006 |
| Asset Index(4Q) | 26.856*** | 30.145*** | 0.061 | 0.074 | 13.808*** | 11.447*** | 0.027 | 0.034 |
| Asset Index(5Q) | 36.843*** | 41.355*** | 0.246 | 0.229 | 18.077*** | 14.986*** | 0.098 | 0.105 |
| Mother education (years) | 1.804*** | 0.831*** | 0.104 | 0.048 | 0.985*** | 0.817*** | 0.049 | 0.057 |
| Mother live with Partner | 4.537*** | 5.092*** | 0.007 | 0.004 | -0.317 | -0.263 | 0.000 | -0.001 |
| Mother’s skin color (white) | -10.896*** | -12.231*** | -0.034 | -0.034 | 6.106*** | 5.062*** | 0.016 | 0.016 |
| Private Health Insurance |  |  |  |  | 8.123*** | 6.734*** | 0.050 | 0.053 |
| **Need: Children's variables** |  |  |  |  |  |  |  |  |
| Sex (female) | -0.370 | -0.415 | 0.000 | 0.000 | 0.515 | 0.427 | 0.000 | 0.000 |
| Very Good Health | 0.532 | 0.597 | 0.000 | 0.001 | 7.733*** | 6.411*** | 0.005 | 0.012 |
| Good Child's Health | -2.082** | -2.336** | 0.006 | 0.005 | 18.288*** | 15.161*** | -0.042 | -0.033 |
| Regular or Bad Child's Health | -3.637 | -4.082 | 0.003 | 0.004 | 35.168*** | 29.156*** | -0.023 | -0.024 |
| Wheezing Chest | 2.744*** | 3.080*** | -0.006 | -0.003 | 6.754*** | 5.599*** | -0.010 | -0.007 |
| Chronic Disease | -0.958 | -1.076 | 0.000 | 0.000 | 2.345** | 1.944** | 0.000 | 0.000 |
| Low Birthweight | 1.206 | 1.353 | 0.000 | 0.000 | -1.140 | -0.945 | 0.000 | 0.000 |
| Hospitalization | 1.259 | 1.413 | -0.002 | -0.001 | 4.171*** | 3.458*** | -0.005 | -0.005 |
| Earache | -1.456** | -1.634** | 0.002 | 0.002 | 5.500*** | 4.560*** | -0.006 | -0.007 |
| Pneumonia | 11.826*** | 13.274*** | 0.000 | 0.000 | 3.756*** | 3.114*** | 0.000 | 0.000 |
| Urinary Infection | 0.599 | 0.673 | -0.007 | -0.009 | -1.610 | -1.335 | -0.002 | -0.002 |
| Breastfeeding (1-3 m) | -1.684 | -1.891 | 0.001 | 0.001 | 4.405*** | 3.652*** | -0.002 | -0.002 |
| Breastfeeding (4-5 m) | 3.566*** | 4.002*** | 0.000 | 0.001 | 1.710 | 1.418 | 0.000 | 0.000 |
| Breastfeeding (6-11m) | 4.155*** | 4.664*** | 0.007 | 0.006 | 1.997 | 1.655 | 0.003 | 0.003 |
| Breastfeeding (>12) | -3.299*** | -3.702*** | 0.004 | 0.002 | 0.209 | 0.174 | 0.000 | 0.000 |
| **Need: Mother's variables** |  |  |  |  |  |  |  |  |
| Mother’s Age (years) | 0.165*** | 0.185*** | 0.005 | 0.004 | 0.146** | 0.121** | 0.004 | 0.004 |
| Smoked in pregnancy | -9.700*** | -10.888*** | 0.028 | 0.026 | 1.557* | 1.291* | -0.004 | -0.004 |
| Very Good Mother's Health | -2.534*** | -2.844*** | -0.005 | -0.005 | -0.156 | -0.129 | 0.000 | 0.000 |
| Good Mother's Health | -2.875*** | -3.228*** | 0.005 | 0.001 | -1.310 | -1.086 | 0.002 | 0.001 |
| Regular or Bad Mother's Health | -7.285*** | -8.177*** | 0.014 | 0.019 | -4.708*** | -3.903*** | 0.007 | 0.009 |
| **Residual** |  |  | -0.008 | -0.049 |  |  | -0.006 | 0.027 |
| **N- observations** | 1,877 | | | | 3,145 | | | |

Notes: *, **, *** denotes p-values less than 10, 5, and 1%. The base groups for asset index, child’s health, breastfeeding, mother’s health are: 1º quintile (1Q), excellent, 0 months, and excellent. Estimations were realized with Generalized Estimating Equation model with log-normal distribution and independent correlation. The PHI expenditures include interaction between income and wave 72 months.

**Table B3**. Contributions of need and non-need variables to CI of total expenditures

|  | **M. Effect-Total Exp.** | | **Contribution-Total Exp.** | |
| --- | --- | --- | --- | --- |
|  | 12M | 48M | 12M | 48M |
| **Non-Need Variables** |  |  |  |  |
| Income | 19.124*** | 16.993*** | 0.208 | 0.197 |
| Asset Index(2Q) | 14.374* | 12.772* | -0.006 | -0.011 |
| Asset Index(3Q) | 30.163*** | 26.802*** | -0.001 | -0.004 |
| Asset Index(4Q) | 47.122*** | 41.871*** | 0.045 | 0.048 |
| Asset Index(5Q) | 60.609*** | 53.855*** | 0.157 | 0.166 |
| Mother education (years) | 3.715*** | 3.301*** | 0.087 | 0.098 |
| Mother live with Partner | -0.815 | -0.724 | -0.000 | -0.001 |
| Mother’s skin color (white) | -0.588 | -0.523 | -0.001 | -0.001 |
| **Need: Children's variables** |  |  |  |  |
| Sex (female) | -4.549*** | -4.042*** | 0.001 | 0.002 |
| Very Good Health | 7.318*** | 6.502*** | 0.002 | 0.005 |
| Good Child's Health | 17.576*** | 15.617*** | -0.019 | -0.015 |
| Regular or Bad Child's Health | 42.672*** | 37.916*** | -0.013 | -0.013 |
| Wheezing Chest | 4.570*** | 4.061*** | -0.003 | -0.003 |
| Chronic Disease | 6.763*** | 6.009*** | -0.000 | -0.000 |
| Low Birthweight | 1.096 | 0.974 | -0.000 | -0.000 |
| Hospitalization | 10.306*** | 9.158*** | -0.005 | -0.006 |
| Earache | 4.452*** | 3.956*** | -0.002 | -0.002 |
| Pneumonia | 3.444 | 3.060 | 0.000 | 0.000 |
| Urinary Infection | -0.216 | -0.192 | -0.001 | -0.001 |
| Breastfeeding (1-3 m) | -1.782 | -1.584 | 0.000 | 0.000 |
| Breastfeeding (4-5 m) | -1.778 | -1.580 | 0.000 | 0.000 |
| Breastfeeding (6-11m) | 2.449 | 2.176 | 0.002 | 0.002 |
| Breastfeeding ( >12) | 1.308 | 1.162 | -0.001 | -0.001 |
| **Need: Mother's variables** |  |  |  |  |
| Mother’s Age (years) | 0.628*** | 0.558*** | 0.007 | 0.007 |
| Smoked in pregnancy | -7.437*** | -6.608*** | 0.009 | 0.008 |
| Very Good Mother's Health | -3.092* | -2.747* | -0.002 | -0.003 |
| Good Mother's Health | -1.999 | -1.776 | 0.001 | 0.001 |
| Regular or Bad Mother's Health | -12.678*** | -11.265*** | 0.010 | 0.011 |
| **Residual** |  |  | 0.009 | 0.031 |
| **N-observations** | 2.509 | | | |

Notes: *, **, *** denotes p-values less than 10, 5, and 1%. The base groups for asset index, child’s health, breastfeeding, mother’s health are: 1º quintile (1Q), excellent, zero month, and excellent. Estimations were realized with generalized estimating equation model with log-normal distribution and independent correlation.

**Figure B1.** Percentage Contribution (%) of variables to CI/EI, by outcome and wave

Notes: The groups correspond to the sum of percentage contributions of variables. **Income** only includes income. **Asset index** only includes asset index. **Health** includes reported health, wheezing chest, chronic disease, low birthweight, hospitalization, earache, pneumonia, and urinary infection. **Mother’s Education** only includes mother’s education. **Need Others** includes breastfeeding, mother’s age, smoked during pregnancy, and sex. **Non-Need Others** includes mother’s race and mother lives with a partner. **PHI** only includes private health insurance. The PHI variable was not included in the decomposition of total expenditures because this variable contains PHI expenditures in its composition.
