# Supplementary material for: Income-related inequality and inequity in children’s health care: A longitudinal analysis using data from Brazil
Source: Soc Sci Med. 2019 Mar;224:127–37. doi: 10.1016/j.socscimed.2019.01.040 (PMC6411923; doi:10.1016/j.socscimed.2019.01.040)
Supplement: C [file mmc3.docx]

**Supplementary document**

In this supplementary document, we seek to show some more detailed results. We also analyze the EI for patient visits (wave 48 months) and the difficulty in obtaining a medical consultation (wave 12 months). Inequality and inequity were observed, and the PHI is a variable with a large contribution to inequality (see table C1). Doctor visits variables were unavailable to longitudinal data.

**Table C1**. Percentage Contribution (%) of variables to CI by outcome

|  | **Patient Visits** | **Difficulty to Medical Consultation** |
| --- | --- | --- |
|  | **48M** | **12M** |
| Income/ Asset Index | 30.84 | 45,15 |
| Health | -44.44 | 17.91 |
| Mother's education | 56.95 | -14.12 |
| Need Others | 8.05 | 14.68 |
| Non-Need Others | 0.64 | -2.53 |
| PHI | 35.74 | 40.30 |
| Residual | 12.22 | -1.37 |
| Erreygers’ Index (EI) | 0,099 | -0,083 |
| Horizontal Index | 0,144 | - |

Notes: The groups correspond to the sum of percentage contributions of variables. **Income/Asset index** includes these two variables. **Health** includes reported health, wheezing chest, chronic disease, low birthweight, hospitalization, earache, pneumonia, and urinary infection. **Mother’s Education** only includes mother’s education. **Need Others** includes breastfeeding, mother’s age, smoked during pregnancy, and sex. **Non-Need Others** includes mother’s race and mother lives with a partner. **PHI** only includes private health insurance.
