# Supplementary material for: Income-related inequality and inequity in children’s health care: A longitudinal analysis using data from Brazil
Source: Soc Sci Med. 2019 Mar;224:127–37. doi: 10.1016/j.socscimed.2019.01.040 (PMC6411923; doi:10.1016/j.socscimed.2019.01.040)
Supplement: D [file mmc4.docx]

**Supplementary document**

In this supplementary document, we seek to show some more detailed results. The table D1 shows the decomposition of the changes between the waves 12 and 24 months in CI, EI, and HI into the two mobility indices $M^{H}$ and $M^{R}$, by outcome (PHI, Medicine use, PHI Expenditures, Medicine Expenditures, and Total Expenditures).  ∆CI, ∆EI, and ∆HI are statistically non-significant.

**Table D1.** Decomposition of changes between 12-24M waves in EI/CI and HI into mobility indices, by outcome

|  | **PHI** | **Medicine Use** | **PHI Expenditures** | **Medicine Expenditures** | **Total Exp.** |
| --- | --- | --- | --- | --- | --- |
| $\Delta CI/\Delta EI$ | 0.017 | -0.016 | -0.007 | -0.031 | -0.011 |
|  | (0.018) | (0.027) | (0.019) | (0.026) | (0.017) |
| $M^{H}$ | -0.007 | 0.016 | 0.034* | 0.034 | 0.023 |
|  | (0.015) | (0.027) | (0.018) | (0.024) | (0.016) |
| $p$ | -0.094 | -0.039 | -0.493 | -0.362 | -0.206 |
|  | (0.883) | (0.071) | (8.312) | (2.895) | (0.223) |
| $q$ | 0.079*** | -0.408*** | -0.068 | -0.094** | -0.111*** |
|  | (0.030) | (0.051) | (0.062) | (0.046) | (0.040) |
| $M^{R}$ | 0.009 | 0.000 | 0.027* | 0.003 | 0.012 |
|  | (0.014) | (0.015) | (0.014) | (0.012) | (0.010) |
| $\Delta HI$ | 0.008 | -0.013 | -0.008 | -0.030 | -0.006 |
|  | (0.018) | (0.028) | (0.019) | (0.026) | (0.017) |
| $M_{HI}^{H}$ | -0.002 | 0.012 | 0.033* | 0.034 | 0.018 |
|  | (0.015) | (0.027) | (0.018) | (0.024) | (0.017) |
| $M_{HI}^{R}$ | 0.006 | -0.002 | 0.026* | 0.004 | 0.012 |
|  | (0.014) | (0.015) | (0.014) | (0.012) | (0.010) |
| N | 2638 | 2638 | 1877 | 3145 | 2509 |

Notes: *, **, *** denotes p-values less than 10, 5, and 1%. ΔEI (for binary variables, PHI and Medicine Use), ΔCI, and ΔHI are respectively variations of EI, CI, and HI. M^H^ and M^R^ are income-related health care mobility and health-related income mobility for each of those cases.*p* represents the progressivity index and *q* is the factor scale. The standard errors of indices are in parentheses and were generated using bootstrapping with 300 replications. The subscript term HI represents the statistics for their respective cases.
